# The effect of pelvic floor muscle training on pelvic floor function and sexuality postpartum. A randomized study including 300 primiparous

**DOI:** 10.1007/s00404-022-06542-z

**Published:** 2022-04-04

**Authors:** Sabine Schütze, Marlen Heinloth, Miriam Uhde, Juliane Schütze, Beate Hüner, Wolfgang Janni, Miriam Deniz

**Affiliations:** 1 Department of Obstetrics and Gynecology, University Hospital of Ulm, Prittwitzstr. 43, 89075 Ulm, Germany; 2 Department of Basic Science, University of Applied Sciences Jena, Jena, Germany

**Keywords:** Pelvic floor muscle training, Primiparous women, Pelvic floor function, Sexuality, Female sexual dysfunction, Female sexual function index

## Abstract

**Purpose:**

Although pregnancy and childbirth are physiological processes they may be associated with pelvic floor disorders. The aim of this study was to evaluate the influence of pelvic floor muscle training on postpartum pelvic floor and sexual function of primiparous.

**Methods:**

This is a randomized prospective study including 300 primiparous women. Due to the dropout 200 women were analyzed. Inclusion criteria were the delivery of the first, mature baby, the ability to speak and understand German. The participants were evaluated by clinical examinations and questionnaires after 6 and 12 months postpartum. After 6 months, the women were randomized in two groups. Compared to the control group the intervention groups participated in 45-min pelvic floor muscle training and pelvic floor perception once a week over 6 weeks.

**Results:**

The results of the questionnaires showed no significant differences between the groups after 12 months. A significant stronger pelvic floor muscle strength was found for the intervention group after 12 months. The improvement of the pelvic floor and sexual function over the time showed a significant improvement in both groups.

**Conclusion:**

Supervised pelvic floor muscle training did not improve both the pelvic floor and the female sexual function in comparison to the control group. After 12 months, the pelvic floor and sexual function improved significant in all women.

**Trial registration:**

German Clinical Trials Register (DRKS00024725), retrospectively registrated.

## Introduction

Although pregnancy and childbirth are physiological processes, they may be associated with potential physical problems. It is known that up to 42% parous women suffer of urinary incontinence (UI), in primiparous 12–35% are affected by flatal incontinence and up to 9.5% are involuntary losing formed stool years after delivery [1]. Besides, two thirds of parous women have anatomical evidence of pelvic organ prolapse [2], however the majority of these women are asymptomatic [3]. Another important topic during and after pregnancy is the change in sexual life. This still seems to be a taboo topic as it is not regularly integrated in the gynecological checkups. Therefore the validated Female Sexual Function Index (FSFI) questionnaire is a great tool to advance research in this topic [4]. Many postpartum problems originate from pelvic floor disorders. The question arises whether pelvic floor muscle training (PFMT) has an influence on the sexual and the pelvic floor function. Martinez et al. 2014 stated, that women with stronger pelvic floor muscles have better sexual function, though the mechanism by which this is achieved is not clearly understood [5]. The systematic review of Ferreira et al. concludes that most studies indicated an improvement of at least one sexual variable due to PFMT [6]. Out of the included studies Citak et al. found positive effects of PFMT 4 months postpartum in primiparous women on the female sexual function as well has on the pelvic floor muscle [7].

The effect of PFMT on urinary and fecal continence postpartum is controversially discussed. The Cochrane analysis of Woodley et al. demonstrated that antenatal conducted PFMT reduced the risk in continent women of becoming incontinent in pregnancy and later. However, PFMT carried out antenatally or postpartum in incontinent women could not prove efficiency [1].

To gain more knowledge of the influence of PFMT on the pelvic floor and sexual function, this randomized study was conducted. The goal was to find ways to help affected women, breaking taboo topics and highlighting the importance of health care workers to speak about these topics.

## Material and methods

### Ethical board approval and registration

This study was approved by the ethics committee Ulm, Germany (377/16) and registered in German Clinical Trials Register (DRKS00024725). Informed consent was obtained from all participants.

### Study design, participants

**Fig. 1:**
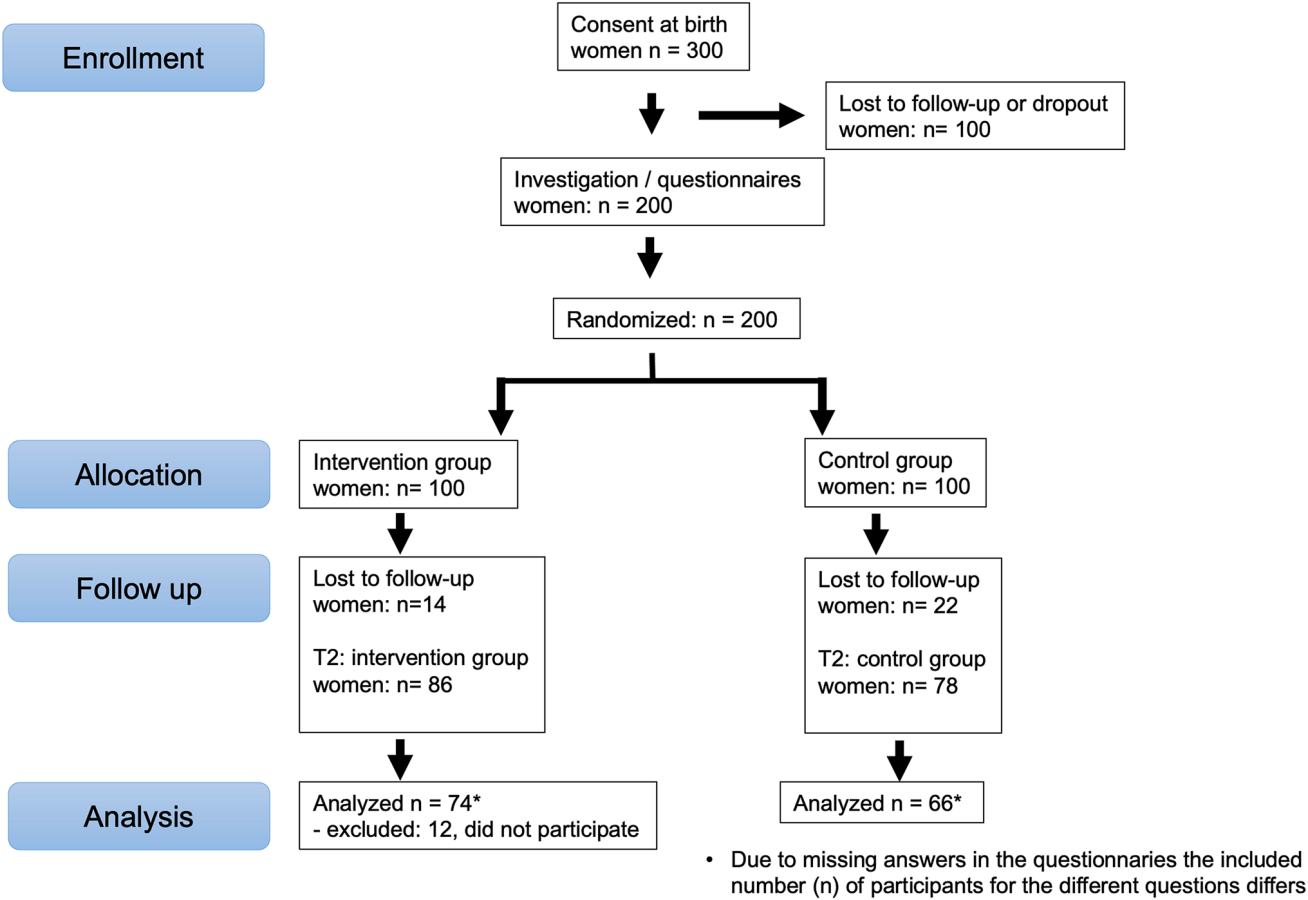
Flow chart of the included participants

All primiparous, who delivered in our hospital between 2018—2019, were asked to take part. Women were informed about the study by a doctor using written and verbal information. Inclusion criteria were primiparous with a term baby and the ability to speak German fluently. Exclusion criteria were secondipara or above, a premature delivery and language barriers. Due to the new approach of this study, no comparable studies were found to carry out a reliable power analysis. Recruitment was stopped when the number of 300 women were included (Fig. 1). The participants were evaluated by examinations and questionnaires (see *Examination*, *Questionnaire)* at 6 (T1) and 12 (T2) months postpartum. The women were instructed to carry out the planed postnatal gymnastic to represent reality. At T1, the women were randomized in two groups (see *Randomization, Intervention*). At T1 200, at T2 164 women participated (Fig. 1). Reasons for the loos to follow-up were moves, limited time, fear of the corona virus, corona infection, other illnesses and no interest. The primary outcome was the effect of PFMT on the FSFI and the Pelvic floor questionnaire for pregnant women and women after childbirth (PFQ). Secondary outcomes were the influence of the PFMT on the grade of prolapse, the pelvic floor muscle contraction and the effect of time between 6 and 12 months.

### Examination

Participants at 6 and 12 months postpartum received questionnaires and underwent a gynecologic examination. This included a speculum examination to evaluate the degree of the prolapse with the pelvic organ prolapse quantification system (POP-Q, [8]). The POP-Q staging is rated from 0 (no prolapse) to 4 (total prolapse). An evaluation of pelvic floor contraction was conducted using the Oxford Score [9]. A modified Oxford Score from 1 (no contraction), 2 (weak), 3 (moderate), 4 (good) to 5 (strong) was used. Four gynecologists specialized in urogynecology performed alternately the examination.

### Questionnaires

Women received the German FSFI-d and the PFQ

#### FSFI-d

The FSFI-d is the German version of the FSFI questionnaire; a 19-item English questionnaire including the domains desire, arousal, lubrication, orgasm, satisfaction, pain. Each domain has a maximum value of 6. The FSFI total score ranges from 2 to 36 [4, 10]. 15 of the items contain a zero option to indicate either “no sexual activity” (12 items) or “did not attempt intercourse” (3 items). The absence of sexual activity or intercourse is not attributable to sexual dysfunction. The incorrect use of the zero category in calculating FSFI domains, except desire, and the total score would underestimate women’s sexual functioning scores [11]. All scores, except desire, were excluded for these women in the analysis. A total score ≤ 26.55 is indicative for female sexual dysfunction (FSD) [12]. Therefore, in both groups, the proportion of FSD was evaluated

#### PFQ

The PFQ consisting of question regarding the delivery, demographic data and 42 specific questions. These specific questions counted in the score and encompassing the four areas of bladder-, anal-, prolapse- and sexual function [13]. The four areas were scaled from 0 (no dysfunction) to 10 (maximal dysfunction). The areas were summated, giving a total score of 0–40.

### Randomization and intervention

A block randomization in two groups, IG—and CG, took place after 6 months. The randomization was prepared in advance with sealed envelopes for a target size of 300 participants. These were opened in the presence of the participants after the examination. The IG comprised weekly, 45-min pelvic floor training for 6 weeks, in groups of ten women instructed by professional pelvic floor physiotherapists. The training included pelvic floor perception based on the Franklin-Method and pelvic floor muscle training. The Franklin-Method uses mental images in combination of physical training. The participants in the IG were asked to do the exercise daily. The CG was asked to do the exercises they had learned in the postnatal gymnastic daily. Out of the IG, 12 women did not take part in the professional pelvic floor training. They were excluded for the statistical analyses.

### Statistical analysis

Statistical analysis was carried out using IBM SPSS Statistics V26. Quantitative data were represented by min, max, median, interquartile range, or mean and standard deviation. Comparisons between IG and CG were analyzed by Man-Whitney *U* or *t*-test, improvement over time between groups was reported by the corresponding differences and analyzed by repeated measurement design. Qualitative data were examined by Chi Square test with Fisher’s exact method in case of expected cell frequency below 5. McNemar test was used for comparison over time. No missing data were imputed resulting in different number of cases in the tests.

## Results

### Study population

Figure 1 shows the number of participants and the lost to follow-up. Table 1 shows the demographic characteristics. The two groups were balanced with no significant differences regarding the demographic factors, examination findings and the questionnaires. Regarding sexual intercourse no significant difference was shown between the groups at T1. The analyses between the lost to follow-ups between T1 and T2 showed no significant difference for demographic factors, examination findings and the FSFI score. The lost to follow-ups had a significant better PFQ total score (*p* = 0.004).

**Table 1:** Demographic data of female participants

Parameters	Intervention group (*n*)	Intervention group (mean/SD)	Control group (*n*)	Control group (mean/SD)	*p*-value
Age in years^a^	100	32.32 (4.70)	100	31.57 (4.56)	0.253
BMI^a^	99	25.38 (6.06)	99	25.57 (4.71)	0.806
Child weight (grams)	100	3297 (402)	100	3332 (523)	0.596
Familiar pelvic floor dysfunction					0.560
Yes	*14*	*14.3%*	*20*	*20%*	
Don’t know	*16*	*16.3%*	*16*	*16%*	
No	*68*	*69.4%*	*64*	*64%*	
Smoking					0.964
Yes	*6*	*6.1%*	*6*	*6%*	
Stopped	*14*	*14.3%*	*13*	*13%*	
No	*78*	*79.6%*	*81*	*81%*	
Self-stated possible pelvic floor contraction					0.349
Yes	*89*	*90.8%*	*91*	*91.9%*	
Don’t know	*5*	*5.1%*	*7*	*7.1%*	
No	*4*	*4.1%*	*1*	*1%*	
Breast feeding					0.965
Never	*7*	*8.0%*	*5*	*6.4%*	
< 6 month	*17*	*19.5%*	*17*	*21.8%*	
> 6 months to 1 year	*39*	*44.8%*	*34*	*43.6%*	
> 1 year	*24*	*27.6%*	*22*	*28.2%*	
PFQ	79	5.08 (2.87)	85	5.16 (3.37)	0.876
FSFI	73	26.45 (4.80)	73	26.2 (5.49)	0.837
POP-Q	98		99		0.517
Oxford-score	98		100		0.410

^a^6 months postpartum

### Intervention

**Fig. 2:**
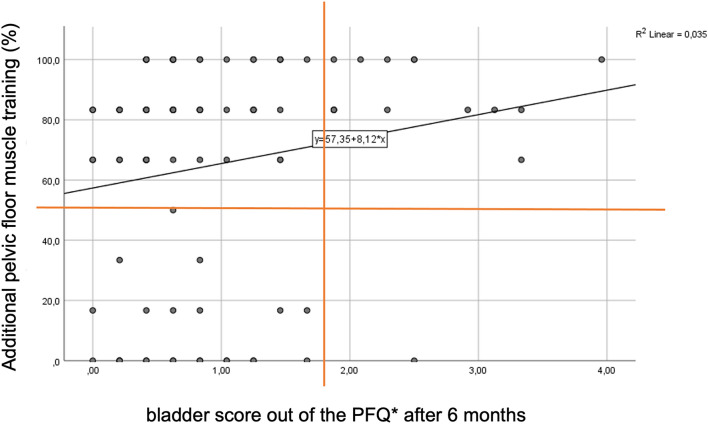
Participants with a bladder score > 1.75 after 6 months out of the PFQ* attended more than 50% of the appointments. Participants out of the interventions group with additional pelvic floor muscle training and a therapy adherence < 50% had a bladder score (with one exception) < 1.75. Significant correlation (*r* = 0.231) between an initially higher bladder score after 6 months and therapy adherence in % (*p* = 0.025). *Pelvic floor questionnaire for pregnant women and women after childbirth

18% of the women in the IG did not take part. 6% of the women joined 17%, 2% joined 33%, 74% joined ≥ 50% of training. Those with $\le$ 50% adherence (with one exception) had a bladder score $\le$ 1.75 at T1. Furthermore, all participants who had a bladder score > 1.75 at T1 attended > 50% of appointments (except the same one exception) (Fig. 2). There was a significant correlation between an initially higher bladder score and therapy adherence (*p* = 0.025; Fig. 2).

### Group comparisons at T2

At T1, 77% of the IG and 73% of the CG had sexual intercourse. At T2, 82% of the women in the control group (CG), 86% of the participants in the intervention group (IG) were included.

Table 2 demonstrates the differences of the FSFI (T2-T1) and the PFQ (T1-T2) over time for each group and compares them. None of the domains were found to be significantly set apart. The POP-Q score showed no significant difference for both groups (IG: *p* = 0.053; CG: *p* = 0.545; group comparison *p* = 0.281). A significant higher Oxford score was found for the IG (IG *p* = 0.005; CG *p* = 0.111; group comparison *p* = 0.018; Table 3). Frequency of sexual intercourse after 12 months showed a significant change in the IG, and a positive trend in the CG. The group comparison was not 55.

**Table 2:** Comparisons of change of the FSFI and the PFQ between the groups after the intervention (12 months; T2)

	Intervention group		Control group		*p*- value
	T2–T1 median [min; max]	*N*	T2–T1 median [min; max]	*N*	
FSFI (total score)	2.7 [− 6.7; 13.8]	44	3.10 [− 5.1; 13.2]	42	0.465
Arousal	0.3 [− 2.4; 3.3]	49	0.6 [− 1.5; 3.0]	45	0.426
Lubrication	0.3 [− 3.0; 3.3]	49	0.3 [− 1.2; 4.8]	45	0.682
Orgasm	0.00 [− 2.8; 3.6]	47	0.4 [− 3.6; 3.2]	44	0.729
Pain	0.4 [− 2; 4]	48	0.6 [0; 4.4]	44	0.775
Desire	0.6 [− 1.2;3.6]	69	0.6 [− 3;2.4]	65	0.432
Satisfaction	0.0 [− 3.6; 4]	49	0.8 [− 4.4; 2.8]	45	0.112

	T1-T2 Median [min; max]	*n*	T1-T2 Median [min; max]	*n*	
PFQ (total score)	1.5 [− 5,1; 9,9]	51	1.1 [− 2,5; 7,6]	57	0.940
Bladder score	0.0 [− 1,5; 3,3]	65	0.0 [− 1,3; 2,1]	63	0.477
Bowl score	0.3 [− 1,3; 2,3]	67	0.3 [− 2,6; 3,6]	65	0.453
Prolapse score	0.0 [− 2; 4]	67	0.0 [− 1,3; 2,7]	76	0.808
Sexuality score	0.4 [− 4,2; 5]	59	0.4 [− 1,3; 4,2]	61	0.633

**Table 3:** Examination findings at the different time points (T1: 6 months and T2: 12 months after delivery) for the respective group as well as the group comparison

	Intervention group			Control group			Group comparison
	T1	T2	*p* value *n*	T1	T2	*p* value *n*	*p* value
POP-Q							
Grade 0	29.4%	48.5%	0.053	35.2%	46.5%	0.545	0.281
Grade 1	61.8%	42.6%	*n* = 68	62.0%	50.7%	*n* = 71	
Grade 2	8.8%	8.8%		2.8%	2.8%		
Oxford-score							
1	11.8%	10.3%	**0.005**	19.4%	11.1%	0.111	**0.018**
2	30.9%	8.8%	*n* = 68	29.2%	16.7%	*n* = 72	
3	22.1%	29.4%		19.4%	34.7% 23.6%		
4	26.5%	27.9%		23.6%	13.9%		
5	8.8%	23.5%		8.3%	11.1%		
Intercourse frequency							
Nor	12.9%	7.1%	**0.002**	11.9%	6.0%	0.086	0.181
Rare	58.6%	42.9%	*n* = 70	49.3%	46.3%	*n* = 67	
Regulary	28.6%	50%		38.8%	47.8%		
Risk for FSD	43.2%	22.7%	**0.049 ***n* = 44	42.9%	11.9%	**0.001 ***n* = 42	0.186

### Improvement over the time

**Fig. 3:**
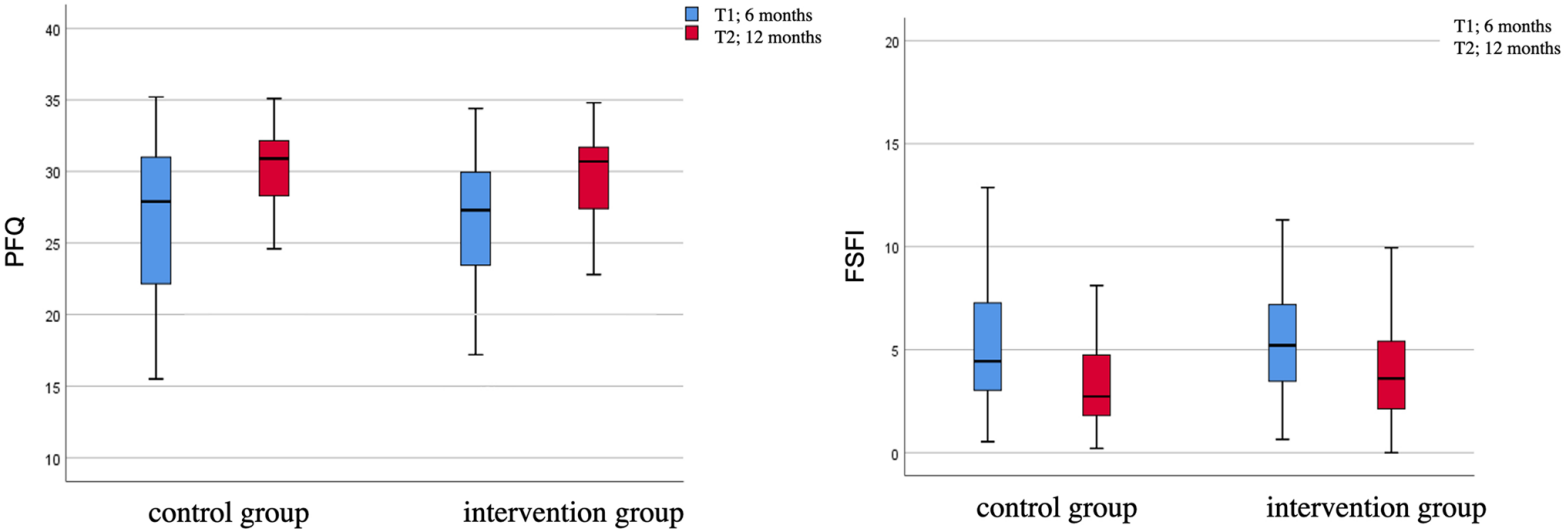
Improvement over time of the Pelvic floor questionnaire for pregnant women and women after childbirth (PFQ) total score on the left side and the Female Sexual Function Index (FSFI) total score on the right side

Both groups showed a significant improvement in the FSFI scores over time. The IG demonstrated a significant improvement over time of the total score (*p* = 0.001), the domains desire (*p* = 0.000), arousal (*p* = 0 0.043), lubrication (*p* = 0.002), orgasm (*p* = 0.010), pain (*p* = 0.000). Only satisfaction was not significant (*p* = 0.153). The following results were found for the CG: Total score (*p* = 0.000; Fig. 3), the domains desire (*p* = 0.000), arousal (*p* = 0.001), lubrication (*p* = 0.000), orgasm (*p* = 0.012), satisfaction (*p* = 0.005), pain (*p* = 0.000). Regarding the question whether there is sexual intercourse no significant difference could be shown over time.

In respect to the PFQ except for the bladder score, all scores were significant improving over the time. IG: total score (*p* = 0.000; Fig. 3), bladder score (*p* = 0.354), anal score (*p* = 0.005), prolapse score (*p* = 0.005), sexual score (*p* = 0.000). CG: total score (*p* = 0.000), bladder score (*p* = 0.051), anal score (*p* = 0.002), prolapse score (*p* = 0.001), sexual score (*p* = 0.000).

Regarding the examination the POP-Q score showed a positive trend over time, however no significant change in both groups and in the group comparison (IG: *p* = 0.053; CG: 0.545; group comparison *p* = 0.281; Table 3). A significant higher Oxford score was found for the IG after 12 months (IG *p* = 0.005; CG *p* = 0.111; group comparison *p* = 0.018; Table 3).

### FSD

The proportion of participants with a FSFI total score ≤ 26.55 did not differ between the groups at T1 (*p* = 0.926). A risk for FSD was identified in 42.9% of CG and 43.2% of IG. After intervention 11.9% of the CG and 22.7% of the IG were indicative for FSD (Table 3). The proportion of participants with FSD did not differ significantly between the groups after intervention (*p* = 0.186). In both groups, there was a significant improvement over time (CG *p* = 0.001, IG *p* = 0.049; Table 3).

## Discussion

The demographic and clinical characteristics were similar in the IG and CG indicating a balanced study population, which is an important foundation to compare these groups. Comparing this collective with the data of Metz et al. a better initial bladder function could be shown, which might be due to the high number of women with the ability to voluntarily contract the pelvic floor muscle [13]. An in-ability to voluntarily contract the pelvic floor muscle goes along with pelvic floor dysfunctions [13].

The main question of this study is the influence of additional PFMT on the sexual and pelvic floor function. Interestingly, participants with a worse bladder score showed a higher therapy adherence. This could be explained due to a higher initial level of suffering.

In the main domains of the FSFI and the PFQ no significant differences could be shown between the groups. This is in accordance with other studies [14–16]. Ahlund et al. randomized 100 primiparous women in home-base PFMT between 3–9 months after delivery. Both groups were re-examined and assessed 9 months postpartum. No differences between these groups were found, both groups showed a significantly better muscle strength after the intervention time [15]. Other studies demonstrated a positive effect of PFMT on the sexual function [6, 17, 18] as well as on the pelvic floor function [16, 19]. The results of our study could be explained by the socio-demographic characteristics of the subjects: highly motivated participants, recruited from a clinic with high socio-economic status. As Baumann et al. stated, both social influences and motivation could be important determinants of adherence to training [20]. It is known, that independent practice in addition to the exercise lessons improves the effectiveness of the postnatal gymnastics [21]. The CG was most likely well motivated and probably adhered to their learned PFMT to the same extent as the IG.

The additional PFMT had a significant effect on the strength of the pelvic floor muscles in this study. However, no association between a stronger pelvic floor muscle and a better sexual function, as demonstrated by Martinez et al., could been shown [5]. It should be mentioned that the Oxford Score, as well as the Ortiz scale used by Martinez et al., are subjectively ascertained parameters [5]. Elenskaia et al. stated that the pelvic floor weakens temporarily after childbirth, but contractility appears to recover by 1 year irrespective of the mode of delivery [22].

The frequency of sexual intercourse over time increased significantly in the IG and tendentiously but not significantly in the CG. However, the comparison between the groups on T2 was not significant. Overall about 50% had regular sexual activity at T2, which highlights the importance of time and the ability to talk about problems regarding this topic during the PFMT.

A significant improvement of pelvic floor and sexual function over the time could be shown in both study groups in almost all domains. This is in line with the studies of Ahlund et al. and Elenskaia et al. [15, 22]. These significant improvement over time can be seen in context with the normal reparative process, the adaption to a live with a baby, the influence of the estrogen level due to 5ation as well as the probably highly motivated participants, who carried out PFMT regularly. This is important for counseling couples antenatally; thus averting many problems in the postnatal period as pelvic floor disorders can have a detrimental effect on a woman’s quality of life.

The bladder score showed no significant improvement over the time. It is known that the strength of the pelvic floor muscles decrease during pregnancy and in the first months after childbirth due to hormonal and anatomical changes, which could lead to UI [23]. The Cochrane analyses of Woodley et al. demonstrated that it is uncertain whether a population-based approach for delivering postnatal PFMT is effective in reducing UI. It should be mentioned that the included studies were not large and most had design problems including limited details to the PFMT, the randomization and poor reporting of the measurements. Uncertainty surrounds the effects of PFMT as a treatment for UI in antenatal and postnatal women, while it is accepted to be effective in mid-life women [1, 24]. Hilde et al. 2013 found, that PFMT does not decrease UI prevalence 6 months after delivery in primiparous women [25]. These data are in line with our findings. The following factors might have an influence. Our study population was not separated for risk factors regarding UI and it seems questionable to compare women after delivery, with those in their mid-life age.

Up to two-thirds of postpartum women have a FSD during the first year after delivery [26, 27]. In this study collective, up to one half of the participants had a risk for FSD, however, after 12 months 22.7% in the IG and 11.9% in the CG suffered from residual FSD. This highlights the importance of time. Our results seem better than those reported in the literature, which may be explained by a better open communication about sexuality in both groups as part of the present study.

Nevertheless, there are limitations. There was a low consistent participation and no control of the regularity of the daily training. A training diary would have been necessary. The CG received no instructions, only the request to continue the exercises as before to simulate the reality. The meta-analysis of García-Sánchez et al. recommends programs which should last 6–12 weeks, with more than three sessions weekly and a length less than 45 min to get the best effect of PFMT on UI [28]. Programs more than three times a week are difficult to implement for mothers with small children. Therefore, the participants were asked to continue doing the learned exercise daily. The clinical examination was conducted by four specialized gynecologists; however, an inter-rater reliability was not raised. Another limitation regards the loos to follow-up, with a high number of participants missing in the final analysis, and the time of the follow-ups. This study should have been ideally carried over a longer time to examine long-term outcome.

In summary supervised PFMT improved the pelvic floor muscle strength but did not improve both the pelvic floor and the female sexual function. Further long-term studies are necessary to evaluate the effect of strict PFMT on the pelvic and sexual function. After 12 months the pelvic floor and the sexual function improved significant in all women highlighting the importance of time in pelvic floor recovery. An open communication about this improvement already antenatally could probably prevent problems for the affected women.

## Abbreviations

FSFI: Female sexual function index
PFQ: Pelvic floor questionnaire for pregnant women and women after childbirth
PFMT: Pelvic floor muscle training
POP-Q: Pelvic organ prolapse quantification system
FSD: Female sexual dysfunction
IG: Intervention group
CG: Control group
UI: Urinary incontinence
